# Assessing the Immunochromatographic Test Strip for Serological Detection of Bovine Babesiosis in Uganda

**DOI:** 10.3390/microorganisms8081110

**Published:** 2020-07-24

**Authors:** Dickson Stuart Tayebwa, Amany Magdy Beshbishy, Gaber El-Saber Batiha, Mariam Komugisha, Byaruhanga Joseph, Patrick Vudriko, Ramadan Yahia, Luay Alkazmi, Helal F. Hetta, Naoaki Yokoyama, Ikuo Igarashi

**Affiliations:** 1 National Research Center for Protozoan Diseases, Obihiro University of Agriculture and Veterinary Medicine, Nishi 2 -13, Inada-cho, Obihiro, Hokkaido 080-8555, Japan; tayebwa.dickson@gmail.com (D.S.T.); amanimagdi2008@gmail.com (A.M.B.); vpato@covab.mak.ac.ug (P.V.); yokoyama@obihiro.ac.jp (N.Y.); 2 RTC Laboratory, College of Veterinary Medicine, Animals’ Resources and Biosecurity, Makerere University, Kampala 7062, Uganda; josephjbvincent@gmail.com; 3 Department of Pharmacology and Therapeutics, Faculty of Veterinary Medicine, Damanhour University, Damanhour 22511, El-Beheira, Egypt; 4 Department of Animal Health, Ministry of Agriculture, Animal Industry and Fisheries, Entebbe 513, Uganda; mariamkomugisha@gmail.com; 5 Department of Microbiology and Immunology, Faculty of pharmacy, Deraya University, Minia 11566, Egypt; ramadanfarrag@rocketmail.com; 6 Biology Department, Faculty of Applied Sciences, Umm Al-Qura University, Makkah 21955, Saudi Arabia; lmalkazmi@uqu.edu.sa; 7 Department of Medical Microbiology and Immunology, Faculty of Medicine, Assiut University, Assiut 71515, Egypt; helal.hetta@uc.edu; 8 Department of Internal Medicine, University of Cincinnati College of Medicine, Cincinnati, OH 45267-0595, USA

**Keywords:** bovine babesiosis, ELISA, ICT, *B. bigemina* C-terminal rhoptry-associated protein (RAP-1/CT17), *B. bovis* spherical body protein-4 (SBP-4)

## Abstract

In Uganda, bovine babesiosis continues to cause losses to the livestock industry because of shortages of cheap, quick, and reliable diagnostic tools to guide prescription measures. In this study, the presence of antibodies to *Babesia bigemina* and *Babesia bovis* in 401 bovine blood samples obtained from eastern and central areas of Uganda were detected using enzyme-linked immunosorbent assays (ELISAs) and immunochromatographic test strips (ICTs). The ELISA and ICT test used targeted the *B. bigemina* C-terminal rhoptry-associated protein (RAP-1/CT17) and *B. bovis* spherical body protein-4 (SPB-4). Using ELISA, single-ICT and dual-ICT, positive samples for *B. bovis* were detected in 25 (6.2%), 17 (4.3%), and 14 (3.7%) samples respectively, and positive samples for *B. bigemina* were detected in 34 (8.4%), 27 (6.7%), and 25 (6.2%), respectively. Additionally, a total of 13 animals (3.2%) had a mixed infection. The correlation between ELISA and single-ICT strips results revealed slight agreement with kappa values ranging from 0.088 to 0.191 between both methods, while the comparison between dual-ICT and single-ICT results showed very good agreement with kappa values >0.80. This study documented the seroprevalence of bovine babesiosis in central and eastern Uganda, and showed that ICT could, after further optimization, be a useful rapid diagnostic test for the diagnosis of bovine babesiosis in field settings.

## 1. Introduction

The agricultural sector is a significant contributor to Uganda’s economy, and the livestock sector plays a key role in the socio-economic well-being of many Ugandans [1,2]. According to statistics from 2011, over 70% of households own livestock, and the livestock sector employs 60% of the rural population [2]. The cattle population in Uganda is estimated at 14 million. Unfortunately, the burden of tick-transmitted diseases, including anaplasmosis, East Coast fever, and babesiosis, has constrained the growth of the cattle population [3,4]. Uganda has a wide variety and distribution of tick species, such as *Rhipicephalus* (*Boophilus*) *decoloratus*, which transmit bovine babesiosis [5,6]. Bovine babesiosis is characterized by fever, anorexia, anemia, jaundice, and hemoglobinuria. Primarily, the appearance of red/dark-colored urine in endemic countries is pathognomonic for bovine babesiosis [7,8]. The treatment of bovine babesiosis at an early stage of infection is based on the administration of imidocarb propionate or diminazene aceturate, at a dosage of 3 mg/kg and 3.5 mg/kg respectively [9,10]. In the late stages of babesiosis infection, blood transfusion may be necessary to resuscitate the patient [11,12]. Following successful treatment, recovered patients can develop lifelong immunity [13,14]. 

Globally, babesiosis is associated with health and economic burdens on livestock production, particularly in Africa, Australia, parts of South and Central America, and Asia [15,16]. Moreover, zoonotic *Babesia* species, namely, *Babesia divergens, Babesia duncani*, and *Babesia microti* (the mouse pathogen)*,* have caused mayhem to humans in parts of Europe and Asia [17]. Although the zoonotic *Babesia* species have not been reported in Africa, the cattle-infecting *Babesia* parasites including *B. bigemina* and *B. bovis* have significant economic impact [15]. It has been estimated that babesiosis has caused a loss of US$50 million per year to cattle farmers in Tanzania [18]. In Uganda, the estimated impact is undetermined but could be worse due to the development of tick acaricide resistance [19]. In a previous study [4], we reported an increase in the prevalence of tick-borne infections in areas affected by acaricide resistance. Prompt diagnosis is therefore a key part of the management strategy for bovine babesiosis. 

Previous studies have shown that diagnostic services are poor in livestock-dominant rural areas of Uganda [4,20]. This has compelled practicing veterinarians and farmers to treat based only on physical examination and experience. Such treatments can lead to the loss of animal lives and further development of resistance to the few anti-babesia drugs available, namely, diminazene aceturate and imidocarb dipropionate [14,16,21]. The high incidence of mixed tick-borne disease (TBD) infections, as previously documented, underlines the urgent need for rapid and precise diagnostic kits to facilitate rational drug prescription and avoid unnecessary losses to farmers [4].

The immunochromatographic test (ICT) strip is a rapid diagnostic serological test that has gained worldwide recognition, as it gives immediate results. In contrast to tests that are more suited to laboratory setups, ICT strips can be used on the farm and results can be obtained in just 15 minutes [22]. The success of ICTs in human medicine compelled researchers to develop test kits for economically impactful animal diseases such as babesiosis. Previous studies documented *B. bovis* diagnostic proteins including the merozoite surface antigen 2 (MSA-2c), rhoptry-associated protein (RAP-1/CT), thrombospondin-related anonymous protein (TRAP-1) and spherical body proteins (SBP-1 and SBP-4) [23,24,25,26]. In comparison, the BbovSBP-4 showed superior antigenic characteristics since it is released at the point when merozoites egress from the red blood cell [27,28,29,30]. On the other hand, the C-terminal truncated rhoptry-associated protein 1 (BbigRAP1/CT17) was tested and used successfully to detect *B. bigemina* infection [27,31]. Later, Kim et al. [27] combined the BbigRAP1/CT17 and *B. bovis* MSA-2c to develop dual ICT strips, but achieved a low viability. Comparatively, Guswanto et al. [31] succeeded by combining BbovSBP-4 and BbigRAP1/CT17 and used the new dual ICT to diagnose *B. bovis* and *B. bigemina* in cattle samples collected from Indonesia. In this study, ICT strips using BbigRAP1/CT17 and BbovSBP-4 were prepared for serological investigation of *B. bigemina* and *B. bovis* in cattle samples collected from Uganda. The results were compared with those obtained using the enzyme-linked immunosorbent assay (ELISA) based on the BbovSBP-4 and BbigRAP1/CT17. 

## 2. Materials and Methods

### 2.1. Study Design

A cross-sectional analysis of sampled bovine blood from eastern and central regions of Uganda was carried out in May and June of 2017. About 4 mL of blood was collected into a red top vacutainer tube (BD Vacutainer^®^, Becton Dickinson and company, Franklin Lakes, NJ, USA) by puncturing the caudal middle vein (tail vein). The vacutainer tubes were inverted gently to homogenize the blood with a clot activator. The vacutainers were packed into an icebox and transferred to the RTC Laboratory at Makerere University. Subsequently, the blood was centrifuged for 5 min at 3000 rpm to obtain serum. Using a pipette, the serum was carefully picked without mixing it with RBC sediment and transferred into 1.5 mL centrifuge tubes. The Eppendorf tubes were packed and transported to the National Research Center for Protozoan Diseases at Obihiro University for further analysis. 

### 2.2. Sample Size Estimation

The sample size was estimated in accordance with the protocol adapted from [32].

n = [(Z_α/2_)^2^ × p (1 − p)]/d^2^ (1)

where n is the required sample size, Z is the desired confidence level (95%) value, p is the anticipated infection prevalence, and d is the required absolute accuracy (tolerable error). In this study, a 5% acceptable error was used; a 50% estimated prevalence was considered since the prevalence for the sampled area was unknown. Based on the calculation, a target sample size of 384 samples was determined, although we collected and analyzed 401 samples.

### 2.3. Preparation of Parasites and B. bigemina RAP-1/CT17 and B. bovis SBP-4 Recombinant Proteins 

The Texas strain of *B. bovis* and the Argentina strain of *B. bigemina* were cultured using a microaerophilic culture system [9,33]. Then, complementary deoxyribonucleic acid (cDNA) was synthesized from these cultures to express *B. bigemina* C-terminal rhoptry-associated protein (RAP-1/CT17) and *B. bovis* spherical body protein 4 (SBP-4) according to the protocol previously described [27,30]. Finally, polyclonal antibodies were obtained by injecting mice with RAP-1/CT17 and SBP-4 recombinant proteins, while the remaining proteins were kept at −30 °C for preparation of ELISA examinations and ICT strips.

### 2.4. Enzyme-Linked Immunosorbent Assay (ELISA)

The standard enzyme-linked immunosorbent assay (ELISA) was conducted in duplicates for each serum sample, following the method defined by Terkawi et al. [30]. Briefly, 50 µL of recombinant antigen (rBbovSBP-4 or rBbigRAP1/CT17) at a final concentration of 0.1 µM in 50 mM carbonate-bicarbonate buffer and pH 9.6 was used to coat the ELISA plates (Nunc 96-well plates, Thermo Fisher Scientific, Austin, TX, USA). The coated ELISA plate was incubated at 4 °C overnight. The plates were then rinsed once with phosphate-buffered saline (PBS) containing 0.05% Tween 20 (PBS-T), and blocked with 100 µL of PBS containing 3% skimmed milk (PBS-SM). The plate was incubated at 37 °C for 1 h. Subsequently, the plates were rinsed once with PBS-T and incubated at 37 °C for 1 h with 50 μL serum samples diluted 1:100 with PBS-SM. After 1 h incubation, plates were rinsed six times with PBS-T and incubated for 1 h at 37 °C with 50 µL of horseradish peroxidase (HRP)-conjugated sheep anti-bovine immunoglobulin G (IgG) (Bethyl Laboratories, Montgomery, TX, USA) as a secondary antibody diluted to 1:4000 with PBS-SM. Plates were further rinsed six times and incubated for 30 min with 100 µL of a substrate solution prepared by mixing 0.3 mg/mL of 2,2’-azide-bis(3-ethylbenzthiazoline-6-sulfonic acid (Sigma, St. Louis, MO, USA), 0.1 M citric acid, 0.2 M sodium phosphate, and 0.01% of 30% H_2_O_2_. An ELISA plate reader (Corona microplate reader MTP-120; Corona, Tokyo, Japan) was used to measure the absorbance at 415 nm after 30 min of incubation at room temperature. Five confirmed negative and positive bovine sera were included on the plate. The cutoff value was determined as the mean value of the optical density at 415 nm for the negative and positive control, plus three standard deviations. All samples were examined twice.

### 2.5. Preparation of the Immunochromatographic Test (ICT) Strips

Immunochromatographic test preparation was performed as previously described, with some modifications [27,31]. Briefly, three ICT strips; *bov*ICT, *big*ICT, and dual-ICTs were prepared based on gold colloid-conjugated recombinant proteins, namely, *B. bigemina* RAP-1/CT17, *B. bovis* SBP-4, and a combination of both as detection antigens. Briefly, 1 mg/mL of rBbovSBP-4 and 1 mg/mL of rBbigRAP-1/CT17 were gently mixed with gold colloids (1:10, *v*/*v*) and incubated for 20 min at room temperature. Then, 0.05% polyethylene glycol (PEG) 20,000 (Sigma-Aldrich, Tokyo, Japan) and 1% bovine serum albumin (BSA; Sigma-Aldrich, Tokyo, Japan) were used to block the conjugated particles that were pelleted after centrifugation for 30 min at 11,200× *g*; the precipitates were then suspended in PBS containing 0.5% BSA and 0.05% PEG. The mixture was further centrifuged and the pellets dissolved in 10 mM Tris-HCl (pH 8.2) with 5% sucrose. Finally, the conjugated gold colloid was soaked in fiberglass paper (Whatmann Standard 17, Whatmann International Ltd., Maidstone, UK) and dried overnight in a vacuum. A BioDot Bio Jet 3050 quanti-dispenser (BioDot, Inc., Irvine, CA, USA) was used to immobilize the recombinant protein and the total IgG onto the nitrocellulose membrane (ImmunoporeRP, GE Healthcare, UK) to create the test and control lines, respectively. The membrane was blocked, washed, and dried for 30 min at 50 °C. Subsequently, the membrane was cut using a BioDot cutter (BioDot Inc., Irvine, CA, USA) into 2-mm-wide strips. 

### 2.6. Evaluation of Performance of the ICT Strips

The ICT test was repeated twice for every sample. Performance evaluation of the ICT strips was conducted on 401 bovine serum samples obtained from the eastern and central areas of Uganda. Twenty microliters of the serum sample were diluted (1:1) in PBS and dropped onto the ICT strip sample pad. The strips were observed for the appearance of bands on the control and test line after 15 min. The results were considered positive only when bands appeared on both the control and test lines, while the results were considered negative when no bands appeared on the test line. The ICT strip was deemed to be invalid if no bands appeared on the control line. 

### 2.7. Statistical Analysis

Epitools-epidemiological data calculators were used to calculate prevalence at a 95% confidence interval (CI 95%) for both the ELISA and ICT generated data. The results generated with ICT were compared to that of ELISA and analyzed with kappa statistic to determine the level of agreement. Agreement was stated to be slight (<0.2), fair (0.21–0.4), moderate (0.41–0.6), good (0.61–0.8), or very good (0.81–1.0) [34].

### 2.8. Ethical Statement

Permission to engage farmers was obtained from the District Veterinary Officer (DVO) of each of the districts involved. Prior to blood sample collection, permission was sought from the farmers. All testing methods were performed in accordance with ethical instructions permitted by the College of Veterinary Medicine Animal Resources and Biosecurity (experiment number: VAB/REC/15/104) and Obihiro University of Agriculture and Veterinary Medicine (Animal experiment number: 280082).

## 3. Results

### 3.1. Seroprevalence of B. bigemina and B. bovis

**Figure 1:**
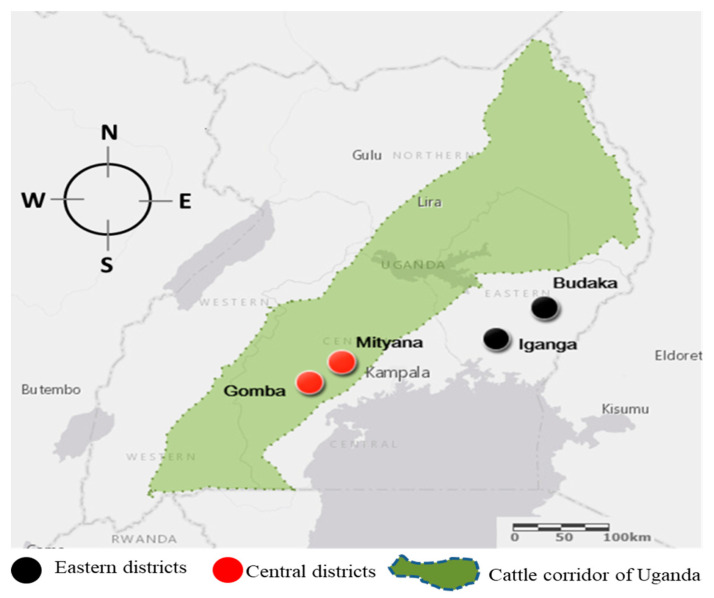
Geographical distribution of the sampling areas. A total of 401 blood samples were obtained from cattle from four locations across Uganda: Gomba (*n* = 105), Buddaka (*n* = 101), Iganga (*n* = 100), and Mityana (*n* = 95).

A total of 401 bovine blood samples were obtained from farms in the eastern and central areas of Uganda (Figure 1), and simultaneously analyzed by ELISA and ICT diagnostics to detect antibodies against *B. bigemina* and *B. bovis*. 

The ICT and ELISA tests detected more antibodies for *B. bigemina* than *B. bovis* (Table 1). All sampling locations had positive samples ranging from 1% to 13.1% (Table 2). The overall prevalence of *B. bigemina* was 8.4%, 6.7%, and 6.2% by means of *big*ELISA, *big*ICT, and dual-ICT, respectively, whereas that of *B. bovis* was 6.2%, 4.3%, and 3.7% by *bov*ELISA, *bov*ICT, and dual-ICT, respectively (Table 1). 

Among all sampling locations, no positive sample of *B. bovis* was detected in Buddaka by *bov*ELISA; however, 1 (1%) serum sample from Iganga was seropositive by *bov*ELISA (Table 2). Mixed infections were detected in a total of 13 cattle (3.2%). 

### 3.2. Comparison between ELISA and ICT Depending on the Kappa Value

The data generated from ELISA, *bov*/*big*ICT, and dual-ICT were examined using kappa statistics in order to determine the agreement between all methods. The kappa values between ELISA with *bov*/*big*ICT and ELISA with dual-ICT ranged from 0.088–0.191 and 0.115–0.173, respectively, indicating slight agreement (Table 3). Meanwhile, the kappa values between *bov*/*big*ICT and dual-ICT ranged from 0.816–0.863, indicating satisfactory agreement (Table 3).

## 4. Discussion

Bovine babesiosis is among the most serious animal diseases in Uganda, and is caused primarily by *B. bigemina* and *B. bovis* [15]. Previous reports in Uganda have used ELISA for the serodiagnosis of bovine babesiosis [35,36,37]. However, this method requires several steps, including coating, blocking, and results are obtained using an ELISA plate reader. In addition, performing the ELISA experiment requires a laboratory setup and trained personnel, which makes it costly [38,39]. Advances in diagnosis led to the introduction of lateral flow-based immunochromatographic test (ICT) strips, which have gained worldwide popularity because they are cheap, give results in 5–30 min, are applicable in both laboratory and field conditions, and do not require a technical person for interpretation of the outcome [39,40]. Since their discovery in the 1970s, the strips have been used for various diagnostic purposes including the detection of human chorionic gonadotropin to diagnose pregnancy in humans [41]. The success of the lateral flow-based ICT attracted many researchers to test its applicability for economically impactful parasites including tick-borne infections. In 2008, Neilsen et al. [42] developed a lateral flow assay targeting the major surface protein 5 (Msp-5) to detect *A. marginale,* while Kim et al. [27] developed and tested a single and dual assay targeting the BbigRAP1/CT17 and BbovMSA-2c proteins for the detection of *B. bigemina* and *B. bovis*, respectively. In subsequent studies, Guswanto et al. [31] combined BbovSBP-4 and BbigRAP1/CT17 to screen babesiosis among samples in Indonesia. In the present study, we used the latter to diagnose *B. bigemina* and *B. bovis* infections among cattle samples collected from Uganda. 

The seroprevalence of *B. bigemina* detected by ELISA, single ICT, and dual-ICT was 8.4%, 6.7%, and 6.2%, respectively; and for *B. bovis* was 6.2%, 4.3%, and 3.7%, respectively. The seroprevalence for *B. bigemina* reported in the current study was low compared to the 26.9% documented by Schischke in 2015. This discrepancy could be due to due the fact that Schischke used a smaller sample size of 130 compared to the 401 samples used in the current study. Alternatively, Schischke conducted her study at the wildlife–livestock interface, where ticks and tick-borne diseases are expected to be high due to the presence of wildlife [3]. The seroprevalence of *B. bigemina* detected in our study was comparable to the PCR prevalence reported in a paper published by our team [4]. On the contrary, *B. bovis* was not detected by PCR, and yet its antibodies were detected by the ICT and ELISA tests used in the current study. The detection of *B. bovis* in an area where *Rhipicephalus* (*B*.) *microplus* had never been reported was startling. However, a report by Muhanguzi et al. [43] complimented our findings after confirming the presence of *Rhipicephalus* (*B*.) *microplus* ticks in south-eastern Uganda. Since *B. bovis* and *Rhipicephalus* (*B*.) *microplus* were already reported in Tanzania, it is likely that the transboundary movement of animals led to their introduction in Uganda [44,45]. 

The agreement between the single-ICT and the dual-ICT kappa score was comparable to that previously recorded by Guswanto et al. [31]. On the other hand, a comparison of the results of either single-ICT or dual-ICT test with ELISA showed slight agreement to detect babesiosis, contrary to the good agreement reported by Guswanto et al. [31]. This detected disparity raises concerns about low sensitivity, specificity, and cross-reactivity, especially in countries where the disease under investigation is endemic [46]. Another possible explanation could be that there is low antibody titer [47] arising from antigenic disparity between the field strains in Uganda and the strains (Argentina strain of *B. bigemina* and the Texas strain of *B. bovis*) used to develop the test kits. Intriguingly, a PCR assay performed on the DNA samples whose serum was used in the current study detected novel *Babesia sp. Mymensingh* in 1% of the samples [48], creating urgency for further epidemiological research and characterization of the antigenic properties of *B. bigemina*, *B. bovis*, and *Babesia sp. Mymensingh* isolated in Uganda. 

## 5. Conclusions

The current study showed that the rBbovSBP-4- and rBbigRAP1/CT17-based single or dual ICT for the detection of bovine babesiosis is a promising rapid diagnostic test for farmers and veterinarians in Uganda. Further optimization is required to improve the sensitivity of the ICT before it can be readily available for commercial use. 

## Figures and Tables

**Table 1:** Screening of *B. bovis* and *B. bigemina* infections in immunochromatographic test (ICT) and ELISA in cattle serum from Uganda.

Parasite Species		*bov*ICT/*big*ICT ^a^	ELISA ^b^		Dual-ICT ^b^		ICT/ELISA ^c^	PCR ^d^
			(+)	(-)	(+)	(-)		
*B. bovis*								
	(+)	17 (4.2%)	3 (0.75%)	14 (3.5%)	13 (3.2%)	4 (1%)	39 (9.7%)	0%
	(−)	384 (95.8%)	22 (5.5%)	362 (90.3%)	1 (0.2%)	383 (95.5%)	362 (90.3%)	
	Total	401	25 (6.2%)	376 (93.8%)	14 (3.5%)	387 (96.5%)	401	
*B. bigemina*								
	(+)	27 (6.7%)	8 (2%)	19 (4.7%)	23 (5.7%)	4 (1%)	53 (13.2%)	13.6%
	(−)	374 (93.3%)	26 (6.5%)	348 (86.8%)	2 (0.5%)	372 (92.8%)	348 (86.6%)	
	Total	401	34 (8.5%)	367 (91.5%)	25 (6.2%)	376 (93.8%)	401	

^a^ Positive (+) and negative (−) samples frequencies as test results of *bov*ICT/*big*ICT. ^b^ Positive and negative samples frequencies as test results of ELISA and Dual-ICT, cross-tabulated with *bov*ICT/*big*ICT test results. ^c^ Positive and negative samples frequencies of combined *bov*ICT/*big*ICT and ELISA test results. ^d^ PCR prevalence of *B. bovis* and *B. bigemina* based on the test results from our previous paper [4].

**Table 2:** The results of ELISA and ICTs of *Babesia bigemina* and *Babesia bovis* in all sampling sites from Uganda.

Sampling Location (District)	No. of Samples	No. of Positive (%)								
		*B. bovis*			*B. bigemina*			Mixed Infection		
		*bov*ELISA	*bov*ICT	Dual-ICT	*big*ELISA	*big*ICT	Dual-ICT	*bov*/*big*-ELISA	*bov*/*big*-ICT	Dual-ICT
Gomba	105	19 (17.7%)	7 (6.7%)	6 (5.7%)	11 (10.2%)	13 (12.4%)	13 (12.4%)	3 (2.8%)	1 (0.9%)	2 (1.9%)
Mityana	95	5 (5.2%)	3 (3.1%)	2 (2.1%)	6 (6.3%)	5 (5.3%)	6 (6.3%)	1 (1.1%)	0 (0%)	0 (0%)
Iganga	100	1 (1%)	2 (2%)	2 (2%)	6 (6%)	2 (2%)	3 (3%)	0 (0%)	0 (0%)	0 (0%)
Buddaka	101	0 (0%)	5 (5%)	4 (3.9%)	11 (10.6%)	7 (6.7%)	3 (2.9%)	0 (0%)	3 (2.9%)	3 (2.9%)
Total	401	25 (6.2%)	17 (4.3%)	14 (3.7%)	34 (8.4%)	27 (6.7%)	25 (6.2%)	4 (1%)	4 (1%)	5 (1.2%)

**Table 3:** Agreement between ICT and ELISA.

Diagnostic Methods	Kappa Value	95% CI ^a^	Agreement ^b^
*bov*ICT and *bov*ELISA	0.088	0.055 to 0.231	Slight
dual-ICT and *bov*ELISA	0.115	0.042 to 0.272	Slight
*bov*ICT and dual-ICT	0.816	0.672 to 0.960	Very good
*big*ICT and *big*ELISA	0.191	0.042 to 0.341	Slight
dual-ICT and *big*ELISA	0.173	0.024 to 0.322	Slight
*big*ICT and dual-ICT	0.863	0.764 to 0.963	Very good

^a^ 95% confidence interval. ^b^ Agreement was examined using kappa statistics and stated as slight (<0.20), fair (0.21–0.40), moderate (0.41–0.60), good (0.61–0.80), or very good (0. 81–1.00) [34].
